# Synthesizing environmentally friendly non-silicone oxygen bleaching stabilizer for linen yarn using oligomeric acrylic acid

**DOI:** 10.1038/s41598-021-89888-9

**Published:** 2021-05-14

**Authors:** Jie Liu, Chun Lv

**Affiliations:** 1 College of Light-Industry and Textile Engineering, Qiqihar University, Qiqihar, 161006 China; 2 College of Architecture and Civil Engineering, Qiqihar University, Qiqihar, 161006 China

**Keywords:** Chemical engineering, Materials chemistry, Process chemistry, Chemical synthesis

## Abstract

Using potassium peroxodisulfate as an initiator and acrylic acid as a monomer, an acrylic acid oligomer was synthesized and then compounded with magnesium salt to form a non-silicone oxygen bleaching stabilizer. By investigating the effects of reaction temperature, reaction time, initiator concentration, monomer concentration, and magnesium salt dosage on product performance, the effect of stabilizers on linen yarn bleaching was analyzed. The synthetic conditions of oxygen bleaching stabilizer were determined by orthogonal test method, namely, acrylic acid monomer concentration 25%, initiator dosage 5%, oligomeric acrylic acid and magnesium salt compound ratio 5:1, reaction temperature 65 °C, reaction time 4 h. At this time, the chelated iron value of the product was as high as 239.314 mg/g, and the chelated calcium value also reached 145.000 mg/g. The dosage of the synthesized stabilizer were determined to be 4 g/L through indicators such as the decomposition rate of hydrogen peroxide and whiteness. The results showed that the environmentally friendly non-silicone oxygen bleaching stabilizer not only had a good ability to inhibit the decomposition of hydrogen peroxide, but also provided bleached linen yarn with a superior degree of whiteness and less metal ion residue, which can effectively solve the “silicon scale” problem and improve the quality of the pre-treatmented products.

## Introduction

Oxygen bleaching stabilizer (OBS) is an essential auxiliary in hydrogen peroxide (H_2_O_2_) bleaching. Hydrogen peroxide bleaching is usually carried out under alkaline conditions, and the trace metals and ions contained in the bleaching solution can catalyze the decomposition of hydrogen peroxide^1–3^. For example, Fe^3+^ can catalyze the ineffective decomposition of hydrogen peroxide, and the hydroxyl radicals generated can attack and destroy the most stable C-H bond when highly activated, causing the fiber brittle. Therefore, oxygen bleaching stabilizer must be applied during hydrogen peroxide bleaching to slow down the rapid decomposition of hydrogen peroxide and protect the fiber from damage^4–6^.

Oxygen bleaching stabilizers mainly include inorganic stabilizers and organic stabilizers. According to its chemical composition, it can be divided into two types: silicon-containing stabilizer and non-silicon-containing stabilizer; According to its component, there are single component and compound type. According to its stability mechanism, there are two kinds: adsorption type and chelating type oxygen bleaching stabilizer^7–9^. The most primary types of oxygen bleaching stabilizers are sodium silicate and phosphonates. Sodium silicate, as an oxygen bleaching stabilizer commonly used in the traditional process, belongs to inorganic adsorption products containing silicon. It has been used in the bleaching process of various textile materials because of its good bleaching effect and low cost. However, it will easily generate insoluble salt with metal ions such as Ca^2+^ and Mg^2+^ during the production process, that is silica scale. The silica scale deposited on the bleaching equipment will bring difficulties to the cleaning work of the equipment. At the same time, the silica scale adhered on the product is easy to cause the fabric rough handle, dyeing unevenly, and even produce white spots and other defects^10–12^. Therefore, the development and application of non-silicon oxygen bleaching stabilizer is increasingly important.

Non-silicon oxygen bleaching stabilizers mainly include phosphonates and macromolecule compounds. Phosphine amino acid, methylene phosphonic acid and ethylenediamine (acid) has been studied most in the past. Christiansen filed a patent for a method for the application of diethylenetriamine pentamethyl phosphonic acid (DTPMPA) in combination with sodium polyacrylate or hydroxyl acrylate polymer as an oxygen bleaching stabilizer^13–15^. Rong-qi Chen^16,17^ developed a phosphonic acid ester type oxygen bleaching stabilizer, which was an excellent stabilizer with both adsorption and chelation functions. Its molecular structure contained oligomers with spatial coordination groups such as phosphoric acid group and hydroxyl group, which was resistant to concentrated alkali, highly concentrated hydrogen peroxide and high temperature. These phosphonates OBS caused water quality deterioration, so not to be recommended to continue development.

Macromolecule stabilizers meet the ecological standardS. Zhu Wenjun et al.^18^ synthesized a polycarboxylic acid with maleic anhydride as the main raw material, and mixed it with an appropriate amount of magnesium chloride to produce OBS capable of both adsorption and chelation. Zhou Lizheng and Luo Kunping^19^ developed a highly efficient non-silicon OBS with strong complexation and adsorption on metal ions. The bleached fabrics felt soft and had good anti-scale effect. Qi Wenyu and Huang Wenbo^20^ developed the OBS FK-601 by polymerizing acrylamide as monomer and then compounding, which eradicated the defects of silica scale adhesion, effectively inhibited the decomposition of hydrogen peroxide and avoided the damage to the fiber. Water-soluble graft polymer including the monomers such as acrylamide, sodium acrylate, (3,4-dihydroxy phenyl) methane sulfonate, N-(trihydroxymethyl) methyl acrylamide, (2-hydroxy phenyl) methane sulfonate has been used as OBS, but the effect applied alone was not better than that in combination with EDTA, DTPA, sodium gluconate, citric acid and quaternary ammonium salt^21–23^. The maleic acid and styrene copolymer, maleic acid and propylene copolymer, polyhydroxycarboxylic acid were used as OBS. There are also modified chitosan synthesis N-(3,5-Diethylamino benzophthalide) chitosan, N-(4-ethylamino benzophthalide) chitosan polymercontaining aromatic amines that can chelate metals, but the cost is very high^24,25^.

In this paper, a type of non-silicon oxygen bleaching stabilizer using acrylic acid and magnesium sulfate as the main raw material was synthesized and applied to the linen yarn. Acrylic acid polymerizes under the action of potassium peroxodisulfate as the initiator to generate oligoacrylic acid, which had good chelating ability and can effectively chelate various metal ions. After compounding with magnesium salt, magnesium hydroxide colloid was formed in the neutral medium, which had strong adsorption capacity. Therefore, the magnesium oligomer acrylate stabilizer can effectively inhibit the catalysis of metal ions, thereby inhibiting the ineffective decomposition of hydrogen peroxide^26^. Experiment results proved that the non-silicon OBS had little damage on textiles materails, and the most important advantage was that the non-silicon OBS has no pollution to the environment and no affinity with fiber, easy to wash out after bleaching, as well as no influences to the bleached products. In addition, this non-silicon OBS is also without phosphorus, meets the environmental requirements. Therefore, synthesis and applications of the synthesized non-silicon oxygen bleaching stabilizer had very vital significance in the bleaching process. It can replace sodium silicate in the pretreatment process and solve the problem of silica scale in the bleaching process.

## Methods

### Materials

The linen yarn used in this study was kindly provided by Qiqihar Jinya Group (China).

### Chemicals

Acrylic acid (chemically pure) as a monomer and potassium peroxodisulfate as an initiator were perchased from Tianjin cameo chemical reagent Co. Ltd. (China). Hydrogen peroxide (30% w/w) as a bleaching agent, magnesium sulfate, ammonium iron (III) sulfate, sodium hydroxide, potassium permanganate, ammonium chloride, ammonia, sulfuric acid, calcium acetate and sodium oxalate were perchased from Tianjin kaitong chemical reagent Co. Ltd. (China). Unless otherwise specified, all the other chemicals used in this study were reagent-grade chemicals.

### Synthesis of non-silicon OBS

This stabilizer was synthesized with solution polymerization. A certain amount of the initiator potassium perdisulfite (2, 3, 4, 5, 6% by the mass of acrylic monomer) and distilled water were added to a three-necked flask containing an electric stirrer, thermometer and reflux condensing unit. Stirring and heating until the initiator potassium perdisulfite was completely dissolved, acrylic acid monomer (10, 15, 20, 25, 30%) and distilled water were added into the initiator solution. Reacted at (50, 55, 60, 65, 70 °C) for (2, 3, 4, 5) h, the light yellow viscous oligoacrylic acid was obtained. Finally added (1, 2, 3, 4, 5 g based on 10 g acrylic acid) magnesium sulfate, continue to stirring for 1 h, then stop the reaction, and non-silicon OBS was obtained.

### Determination of chelated iron value

The stabilizer solution was titrated with a standard Fe^3+^ solution. The titration end point was when the stabilizer solution appeared turbidity or color change. The ability to chelated iron ions was expressed in milligrams of chelated trivalent iron ions per gram of the stabilizer. Chelated iron value was calculated by Eq. (1).

1 $$ {\text{Chelated}}\,{\text{iron}}\,{\text{value}} = V/(G \times 10/100) $$

where V is the volume of 1.0 g/L standard Fe^3+^ solution consumed (ml), G is the weight of the stabilizer used (g).

### Determination of chelated calcium value

The stabilizer solution was titrated with calcium standard solution, using sodium oxalate as an indicator. The titration end point was when the stabilizer solution appeared white calcium oxalate precipitation. Chelated calcium value was calculated by Eq. (2).

2 $$ {\text{Chelated}}\,{\text{calcium}}\,{\text{value}} = (V \times C \times 100)/(G \times 25/100) $$

where V is the volume of the calcium acetate solution consumed (ml), C is the concentration of the calcium acetate solution (mol/L), G is the weight of the stabilizer used (g).

### Determination of decomposition rate of hydrogen peroxide

The decomposition rate (DR) of H_2_O_2_ was determined with the stanard solution of potassium permanganate and calculated by Eq. (3).

3 $$ DR = (V_{0} - V_{1} )/V_{0} \times 100\% $$

where V_0_ is the volume of the standard solution of potassium permanganate consumed by 5 ml of bleaching bath before bleaching, V_1_ is the volume of the standard solution of potassium permanganate consumed by 5 ml of bleaching bath after bleaching^27–29^.

### Whiteness measurement

Refer to Textile Fiber Whiteness Chroma Test Method GB/T 17644-2008 for determination. The linen roving yarns was measured on a spectrophotometer (YQ-Z-48A whiteness color tester, China) using a D_65_ illuminant and 10° standard observer. Each sample was about 80 mm long and was closely arranged on the pallet of the whiteness color tester. Each sample was rotated in four different directions for measurement. An average of four readings was calculated for each sample.

## Results and discussion

### Synthesis of non-silicon oxygen bleaching stabilizer

#### Effect of acrylic monomer concentration on chelated value

**Figure 1:**
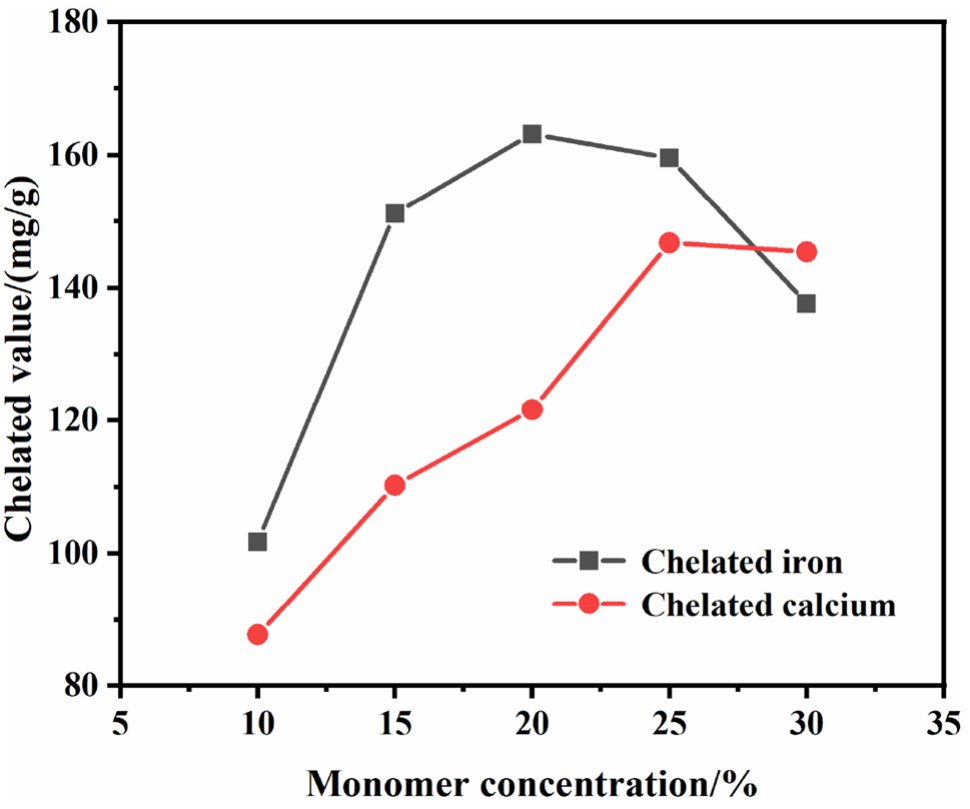
Effect of acrylic monomer concentration on chelated value.

In this experiment, water was selected as the solvent, the monomer was 10 g acrylic acid, the amount of distilled wate was added to change the acrylic monomer concentration upto 10, 15, 20, 25, 30%, respectively. Potassium persulfate dosage was 4% (2.5 g), reacted at 70 °C for 4 h, then added 2 g magnesium sulfate, continued to react for 1 h. The effect of acrylic monomer concentration on chelated value is shown in Fig. 1.

It can be seen from Fig. 1, the chelated value increased gradually with increasing of monomer concentration at the beginning. The reason is that with increasing of monomer concentration, the polymerization rate accelerated, the monomer conversion rate improved, and polymers amount increased, that is, the chelating ability was gradually enhanced. The monomer concentration increased to 20%, the chelated iron value reached its maximum level, and increased to 25%, the chelated calcium value reached its maximum, but the chelated iron value decreased. After that, the chelated iron value and chelated calcium valuehe decreased with the monomer concentration increased. It indicates that appropriate acrylic acid used was beneficial to the chelating effectiveness. When acrylic acid monomer added too much, the molecular weight of the polymerized products increased, the viscosity of the products also increased and leaded to operate difficultly, thus the chelating effect decreased. In view of the reason that Fe^3+^ had stronger effect on accelerating the decomposition of H_2_O_2_, the concentration of acrylic acid should be about 20%.

#### Effect of magnesium sulfate dosage on chelated value

**Figure 2:**
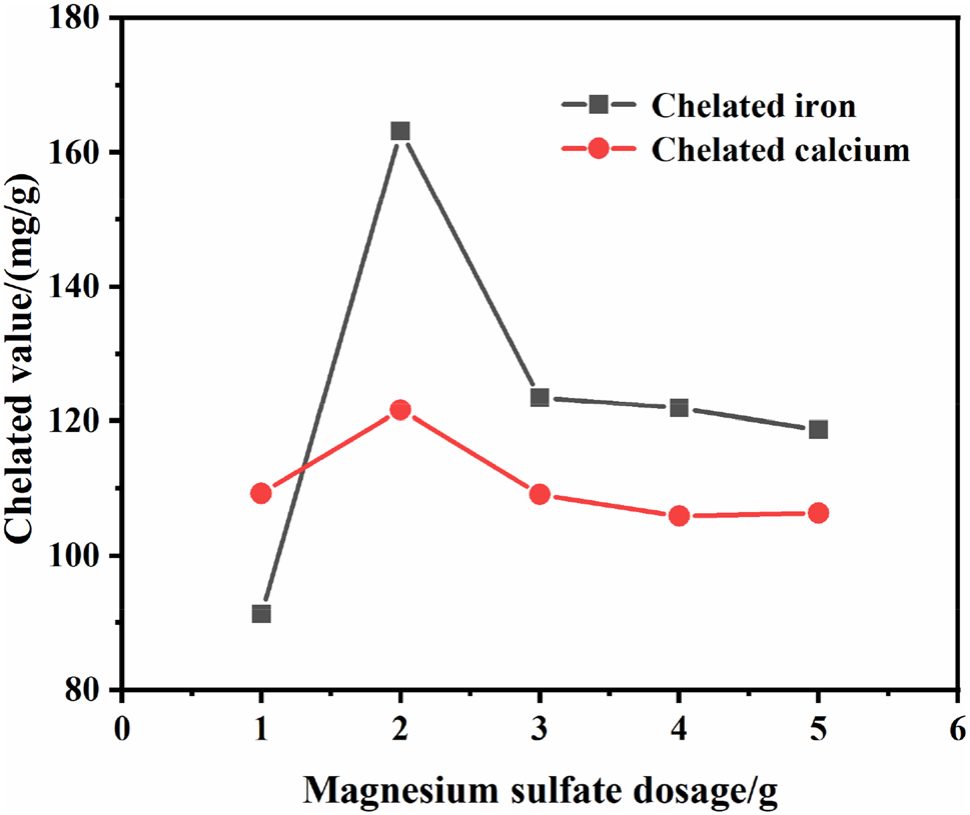
Effect of magnesium sulfate dosage on chelated value.

The concentration of acrylic monomer was 20% (10 g acrylic acid and 40 g distilled water), potassium persulfate was 4% of the mass of acrylic acid used, reacted at 70 °C for 4 h, then added 1, 2, 3, 4, 5 g magnesium sulfate respectively, continued to react for 1 h. The effect of magnesium sulfate dosage on chelated value is shown in Fig. 2.

It can be seen from Fig. 2, the magnesium sulfate dosage was between 1 and 2 g, the chelated iron value increased from about 90 mg/g to about 160 mg/g. The reason is that magnesium salt existed in the form of magnesium hydroxide micelles in the alkaline medium, while magnesium salt dosage increased, the number of generated micelles increased and enhanced the adsorption capacity. After that, the chelated iron value and chelated calcium valuehe decreased with the magnesium sulfate dosage increasing. The reason is that excessive magnesium salt leaded to an increase in the number of magnesium hydroxide micelles with positive charges, and the carboxyl groups with negative charges on the polymer chain were adsorbed and chelated, thus reduced the number of effective carboxyl negative ions and affected the chelation of positive metal ions by carboxyl negative ions^30,31^. Although the change of chelated calcium value was not as obvious as that of chelated iron value, it also reflected the same trend. The optimal magnesium sulfate dosage should be 2 g, that is, the mass ratio of acrylic acid to magnesium sulfate should be 5:1.

#### Effect of temperature on chelated value

**Figure 3:**
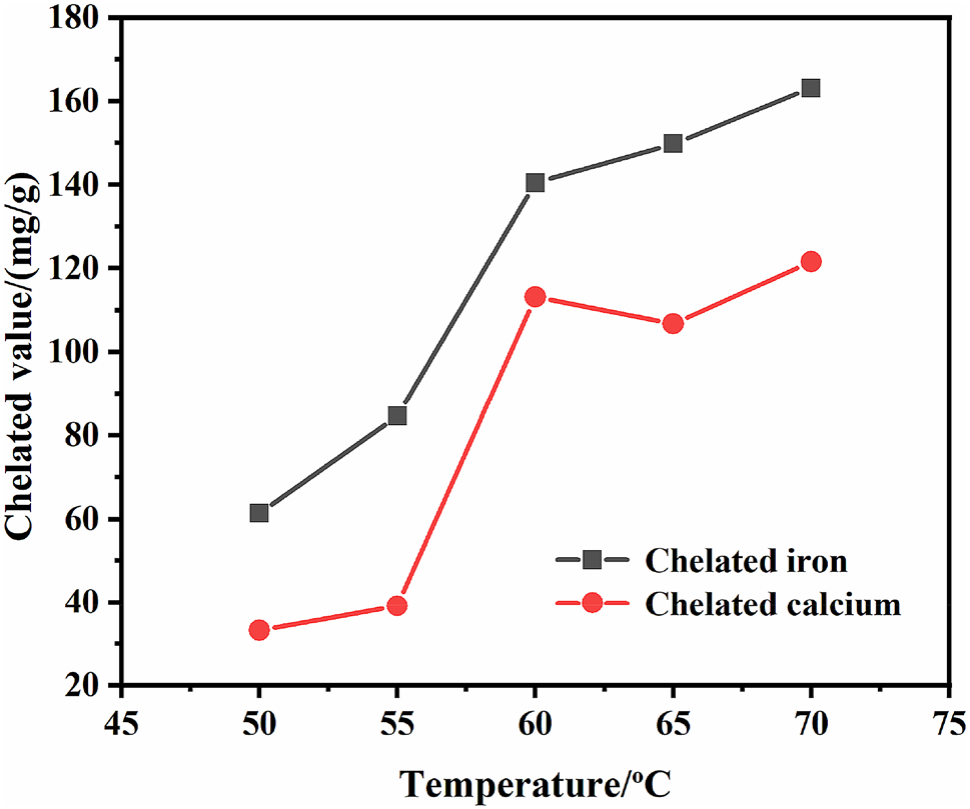
Effect of temperature on chelated value.

The acrylic monomer concentration was 20%, potassium persulfate dosage was 4%, respectively reacted at 50, 55, 60, 65, 70 °C for 4 h, then added 2 g magnesium sulfate, continued to react for 1 h. The effect of temperature on chelated value is shown in Fig. 3.

It can be seen from Fig. 3, the chelated value increased gradually with temperature rised. With increasing of the polymerized temperature, the movement of each molecule in the reacting system was accelerated, and the collision probability between molecules increased. The polymer viscosity became larger and larger, indicating that the molecular weight of the polymer also increased. The chelating ability was improved by the formation of more polymers^32^. When the temperature continued to rise to 75 °C, the reactants was too viscous to obtain the final product. So the temperature of synthetic reaction should be at about 70 °C.

#### Effect of initiator dosage on chelated value

**Figure 4:**
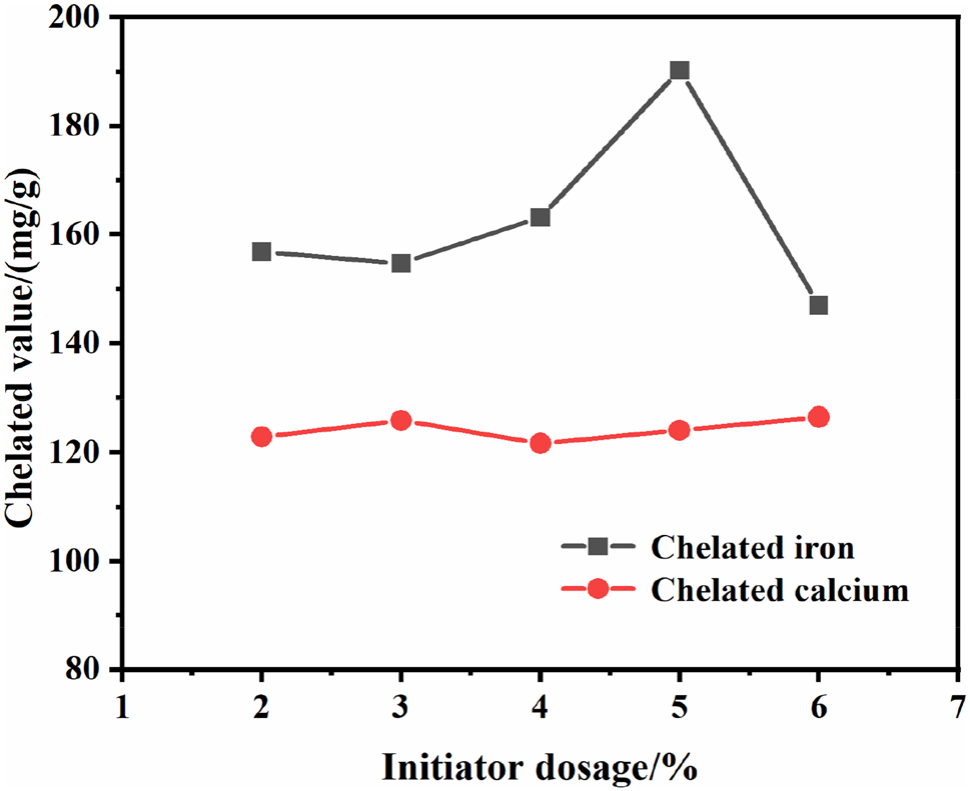
Effect of initiator dosage on chelated value.

The concentration of acrylic monomer was 20%, the dosage of potassium persulfate as initiator was 2, 3, 4, 5, 6% by the mass of acrylic monomer, respectively, reacted at 70 °C for 4 h, then added 2 g magnesium sulfate, continued to react for 1 h. The effect of initiator dosage on chelated value is shown in Fig. 4.

It can be seen from Fig. 4, when the potassium persulfate dosage increased from 2 to 4%, the chelated value changed gently. The reason is that the initiator dosage was too little to generate a lot of free radicals, the chain initiation efficiency was lower, the monomer conversion rate was also lower, and the effective chelate components were few. When potassium persulfate increased to 5% of the mass of acrylic acid, the chelated iron value reached the maximum, it indicated that the amount of initiator met the requirements of the polymerization, and the number of free radicals generated was enough to initiate the monomer polymerization. The potassium persulfate dosage increased to 6%, the chelated iron value decreased significantly, indicated that excessive initiator increased the crosslinking degree of monomers, decreased the sensitivity and reduced the chelating performance^33^. The chelated calcium value hardly fluctuated, indicating that initiator dosage had little effect on chelated calcium value. Therefore, the most suitable dosage of potassium persulfate should be 5% of the mass of acrylic monomer.

#### Effect of reaction time on chelated value

**Figure 5:**
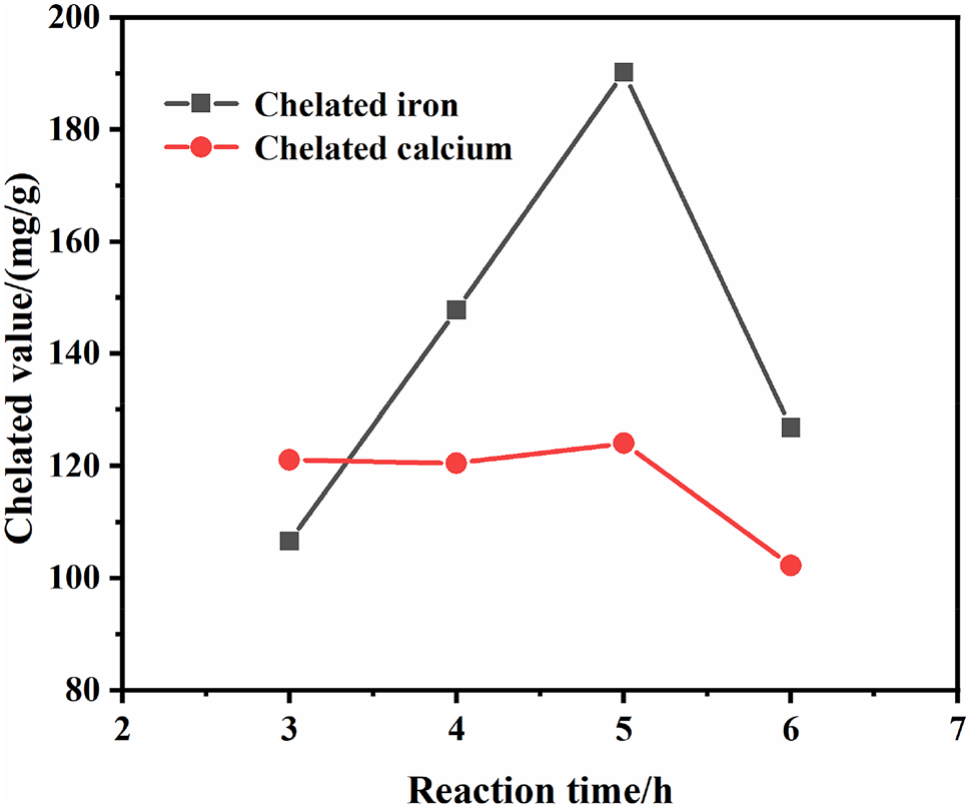
Effect of reaction time on chelated value.

The concentration of acrylic monomer was 20%, potassium persulfate dosage was 4%, reacted at 70 °C for 2, 3, 4, 5 h, respectively, then added 2 g magnesium sulfate, continued to react for 1 h, so the total reaction time was 3, 4, 5, 6 h. The effect of reaction time on chelated value is shown in Fig. 5.

It can be seen from Fig. 5, in the range of 3–5 h, the chelated iron value showed an upward trend and increased greatly, reached the maximum value at 5 h, while the chelated calcium value almost did not fluctuate in this range. However, when the total reaction time increased to 6 h, the chelated iron value and chelated calcium value decreased significantly, so the optimal reaction time should be 5 h. The reason is that the reaction time was too short to polymerize completely, and leaded to uneven distribution of molecular weight of the polymerization products, affecting the performance of the final products. On the contrary, the reaction time was too long, the polymerized molecular chain segments continued to polymerize, resulting in a burst polymerization, which increased the molecular weight of the product and also affected its chelating performance.

#### Orthogonal optimization of synthesis process of non-silicon OBS

Based on the influences of single factor on the chelated effect of the stabilizer, the optimized synthesis process of non-silicon OBS was determined by orthogonal experiment of L_16_ (4^5^). The result is shown in Table 1, and the range analysis is shown in Table 2.

**Table 1:** The orthogonal Layout.

No	*A*(MgSO_4_)/g	*B*(Acrylic Con.)/%	*C*(Initiator)/%	*D*(Temperature) /℃	*E*(Time)/h	Chelated iron value/(mg/g)	Chelated calcium value/(mg/g)
1	1(1)	10(1)	4.0(1)	60(1)	4.0(1)	90.071	32.610
2	1(1)	15(2)	4.5(2)	65(2)	4.5(2)	116.285	100.312
3	1(1)	20(3)	5.0(3)	70(3)	5.0(3)	130.273	104.441
4	1(1)	25(4)	5.5(4)	75(4)	5.5(4)	143.569	140.896
5	1.5(2)	10(1)	4.5(2)	70(3)	5.5(4)	75.859	62.396
6	1.5(2)	15(2)	4.0(1)	75(4)	5.0(3)	99.855	94.762
7	1.5(2)	20(3)	5.5(4)	60(1)	4.5(2)	136.319	119.505
8	1.5(2)	25(4)	5.0(3)	65(2)	4.0(1)	193.126	138.606
9	2.0(3)	10(1)	5.0(3)	75(4)	4.5(2)	119.781	89.264
10	2.0(3)	15(2)	5.5(4)	70(3)	4.0(1)	124.008	98.959
11	2.0(3)	20(3)	4.0(1)	65(2)	5.5(4)	168.725	128.614
12	2.0(3)	25(4)	4.5(2)	60(1)	5.0(3)	166.505	137.087
13	2.5(4)	10(1)	5.5(4)	65(2)	5.0(3)	136.072	76.110
14	2.5(4)	15(2)	5.0(3)	60(1)	5.5(4)	101.748	91.174
15	2.5(4)	20(3)	4.5(2)	75(4)	4.0(1)	158.185	127.156
16	2.5(4)	25(4)	4.0(1)	70(3)	4.5(2)	176.589	145.103

**Table 2:** Analysis of orthogonal experimental results.

	A	B	C	D	E	
Chelated iron value	K_1_	480.198	421.783	535.240	494.643	565.390
	K_2_	505.159	441.896	516.834	614.208	548.974
	K_3_	579.019	593.502	544.928	506.729	506.873
	K_4_	572.594	679.798	539.968	521.390	489.901
	k_1_	120.0495	105.4458	133.8100	123.6608	141.3475
	k_2_	126.2898	110.4740	129.2085	153.5520	137.2435
	k_3_	144.7548	148.3755	136.2320	126.6823	126.7183
	k_4_	143.1485	169.9473	134.9920	130.3475	122.4753
	R	24.7053	64.5015	7.0235	29.8912	18.8722
Chelated calcium value	K_1_	378.259	260.380	401.089	380.376	397.331
	K_2_	415.269	385.207	426.951	443.642	454.184
	K_3_	453.924	479.716	423.485	410.899	412.400
	K_4_	439.543	561.692	435.470	452.078	423.080
	k_1_	94.5648	65.0950	100.2723	95.0940	99.3328
	k_2_	103.8173	96.3018	106.7378	110.9105	113.5460
	k_3_	113.4810	119.9290	105.8713	102.7248	103.1000
	k_4_	109.8858	140.4230	108.8675	113.0195	105.7700
	R	18.9162	75.3280	8.5952	17.9255	14.2132

It can be seen from Tables 1 and 2, for the chelated iron value, the order of primary and secondary factors was B > D > A > E > C, the optimization scheme was A_3_ B_4_ C_3_ D_2_ E_1_. For the chelated calcium value, the order was B > A > D > E > C, the optimization scheme was A_3_ B_4_ C_4_ D_4_ E_2_. Factor B was the first major factor, for two indicators of chelated iron value and chelated calcium value, the optimal level was B_4_, so B_4_ was selected. Factor A was the third main factor for chelated calcium value and was the second main factor for chelated iron value, the optimal level was A_3_, so A_3_ was selected. Factor D was the second main factor for chelated iron value, D_2_ was the best; but for chelated calcium value, D_4_ was the best. In view of the difficulty of polymerization at 75 °C in single factor test, D_2_ was selected. Factor E was the fourth major factor. For the chelated iron value, E_1_ was the best, the reaction time was 4 h, and for chelated calcium value, E_2_ was the best (4.5 h). Because of the more important influenced the chelated iron value than the chelated calcium value on this experiment, and the reason of energy saving, E_1_ was selected. Factor C was the secondary influencing factor. For the chelated iron value, C_3_ was the best, and for the chelated calcium value, C_4_ was the best, because factor C had very little affect on both indicators, so C_3_ was selected to save the cost. So the final synthetic process of non-silicon oxygen bleaching stabilizer was A_3_ B_4_ C_3_ D_2_ E_1,_ that was, the dosage of magnesium sulfate 2 g (the mass ratio of acrylic acid and magnesium sulfate was 5:1), the concentration of acrylic monomer 25%, the dosage of initiator 5% of the mass of acrylic monomer, the reaction temperature was at 65 °C and the total reaction time was 4 h.

The optimized process determined by orthogonal experiment was used for verification experiments, and the properties of the product were determined. The results showed that the chelated iron value was as high as 239.314 mg/g, and the chelated calcium value was as high as 145.000 mg/g, which was much higher than any one of the results on single factor and orthogonal experiments, so the synthesis process of the non-silicon oxygen bleaching stabilizer was feasible.

#### Mass spectra analysis

**Figure 6:**
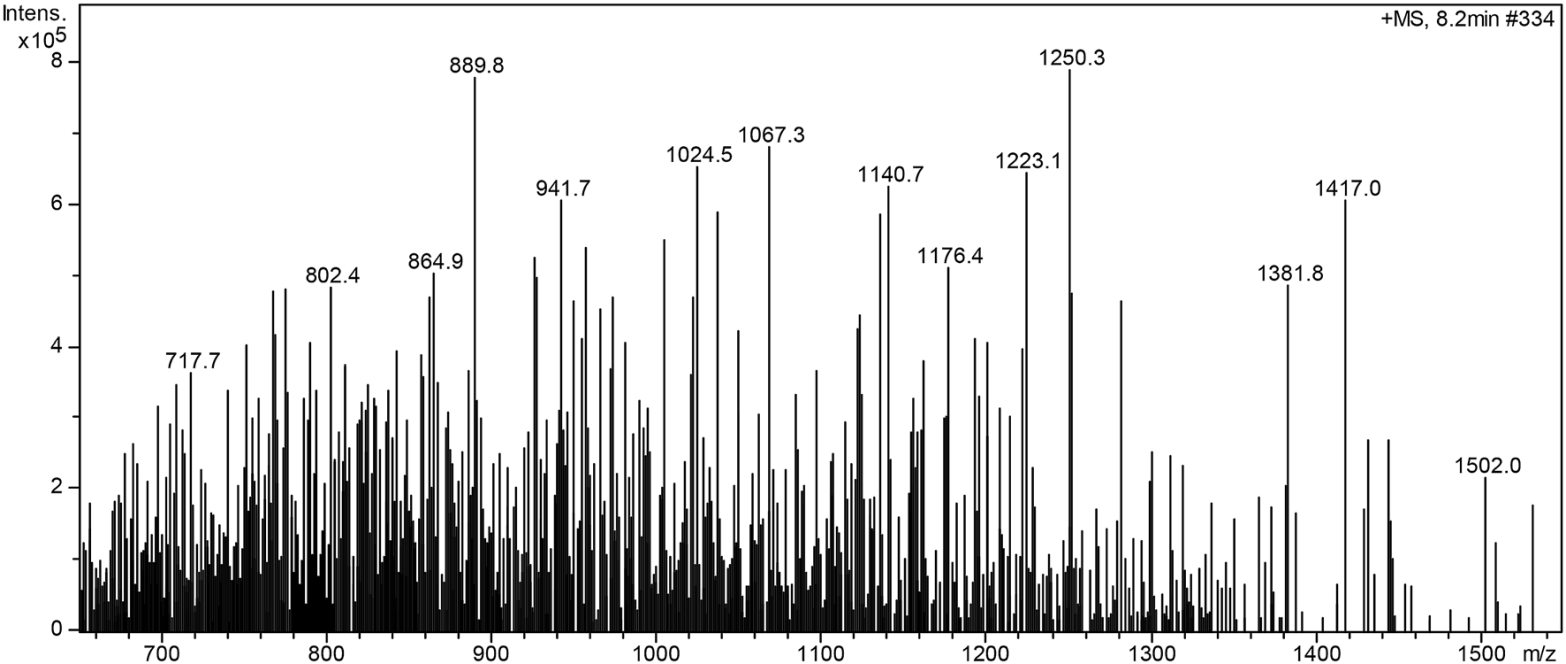
MS figure of the non-silicon oxygen bleaching stabilizer.

To determine the molecular weight of the product and its distribution, the mass spectrometer of 6310 Ion Trap LC–MS by Agilent Corporation (USA) was used. As shown in Fig. 6, the molecular weight of the product was distributed in the range of 700–1500, the main range was in 890–1250. The figure showed that the highest peaks were at 889.8 and 1250.3, it indicated that the molecular weight of the product was mainly distributed in this range, and there were more strong peaks within this range, such as 941.7, 1024.5, 1067.3, 1140.7, 1223.1, etc., indicated the molecular weight distribution was relatively uniform, ranging from 890 to 1250.

#### Infrared spectra analysis

**Figure 7:**
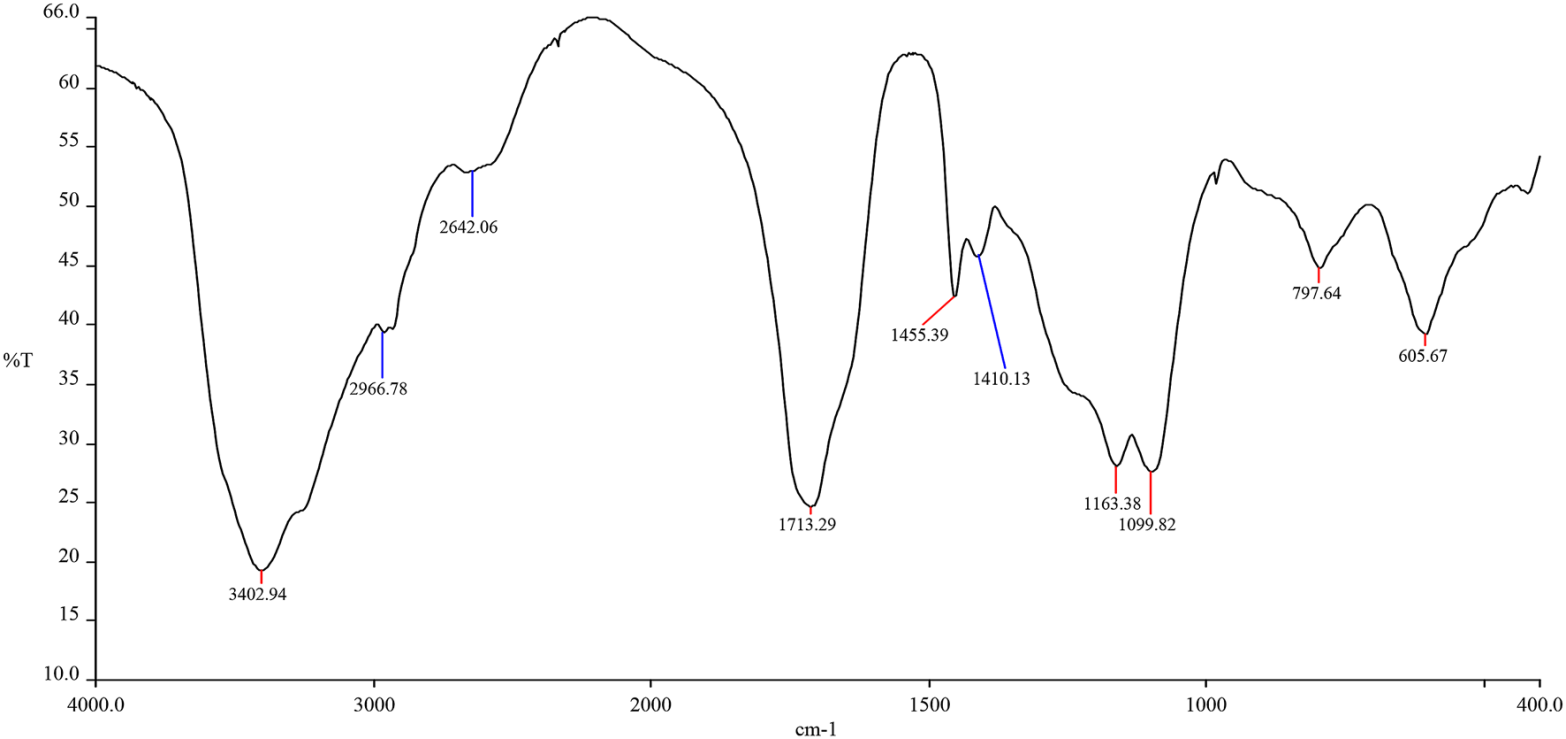
IR Figure.

Spectrum One Fourier Transform Infrared Spectrometer by PerkinElmer (USA) was used. The infrared spectrum of synthesized non-silicon oxygen bleaching stabilizer was shown in Fig. 7.

Bands at around 1700 cm^−1^ were assigned to poly(acrylic acid). In Fig. 7, the peak observed at 1713.29 cm^−1^ was due to C=O stretching vibration in poly(acrylic acid), at 1163.38 cm^−1^ was due to C-O stretching vibration in poly(acrylic acid). The strong band was found at around 1710 cm^−1^ and a characteristic band was found at 1450 cm^−1^, it indicated that polyketones was producd. The narrow absorption peak observed at 1435.39 cm^-1^ was assigned to the presence of a trace polyketone. A broad absorption peak observed at 3402.94 cm^−1^ indicated that hydrogen bonds were formed between the molecules in the product, including the formation of hydrogen bonds between molecules, two-molecular associations, or multimolecular associations^34^. The weaker absorption peaks observed at 2966.78 and 2642.06 cm^−1^ indicated that O–H and other groups in the molecule formed hydrogen bonds, conduciving to form chelating bonds for increasing the chelating performance of the product. In addition, the absorption peaks observed at 797.64 and 605.67 cm^−1^ indicated that the product contained substituted benzene, unsaturated double bonds, which may be the reason that the product contained less unreacted acrylic monomer. Infrared spectrum showed that the target product was obtained.

### Determination of the synthesized stabilizer dosage in bleaching linen yarn

#### Effect of the stabilizer dosage on decomposition rate of H_2_0_2_

**Figure 8:**
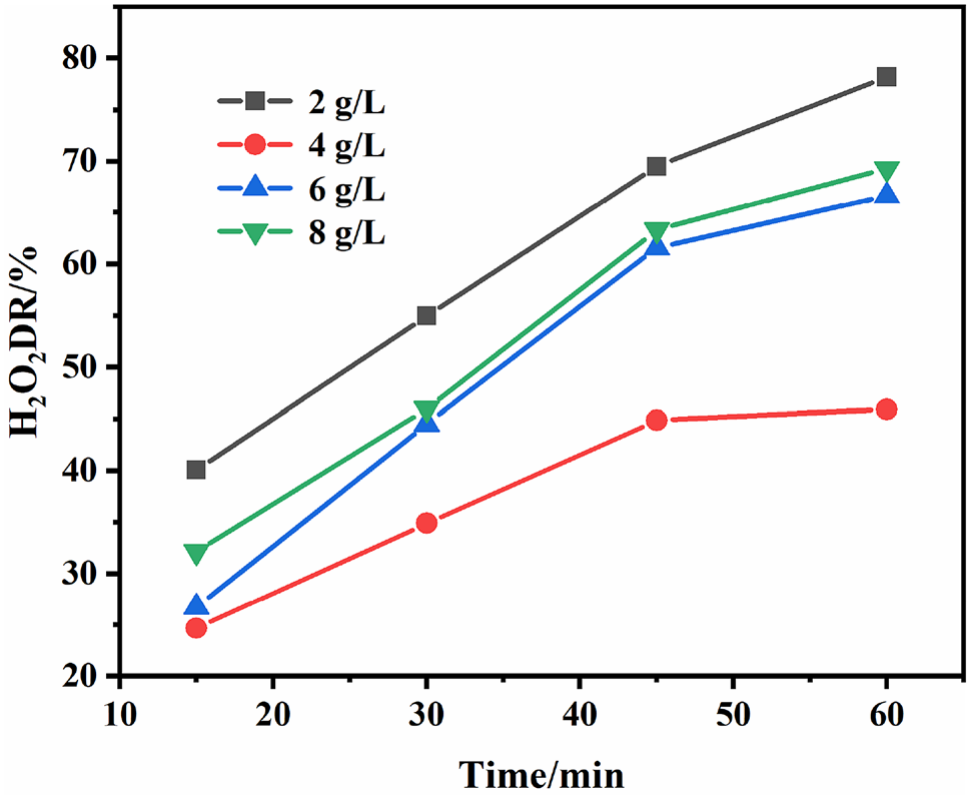
Effect of the synthesized stabilizer on H_2_O_2_ decomposition rate.

In hydrogen peroxide bleaching, stabilizer must be added to inhibit the excessive decomposition of hydrogen peroxide and avoid severe damage to the yarn or fabric. And the dosages of the stabilizer may affect the decomposition rate of hydrogen peroxide. The bleaching process was carried out with a bath ratio of 1:20. Each bleaching solution contained H_2_0_2_ 5 g/L, Na_2_CO_3_ 2.5 g/L, NaOH 1 g/L, wetting agent JFC 1 g/L, stablizer (2, 4, 6, 8 g/L), bleaching at 90–95 °C for 60 min. The linen yarn was 10 g for each experiment. The decomposition rate of hydrogen peroxide under different stabilizer dosages was measured every 15 min. The average value was taken from the two bleaching tests. The effect of the synthesized stabilizer on H_2_O_2_ decomposition rate is shown in Fig. 8.

From the results shown in Fig. 8, when 2 g/L synthesized stabilizer were added in the bleaching bath, the decomposition rate of H_2_O_2_ was the highest at any time, it was closed to 80% at bleaching 60 min. The reason was that the stabilizer dosage was too small to inhibit the decomposition of H_2_O_2_. When the stabilizer was added to 4 g/L, the decomposition rate of H_2_O_2_ decreased significantly by as much as 20%. The decomposition rate of H_2_O_2_ was 24.67% at bleaching 15 min, and did not exceed 50% at the end of bleaching 60 min. At this time, the stabilizer dosage was relatively moderate. The stabilizer dosage in bleaching bath was not high, and the polymer chains can be diastolic, which can adsorb and chelate the catalytic material well, and effectively reduced the ineffective decomposition of H_2_O_2_. While it increased to 6 g/L, the decomposition rate of H_2_O_2_ did not continue to reduce. The stabilizer were added to 8 g/L, the decomposition rate was not continuing to reduce. It seems that increasing the amount of stabilizer can no longer reduce the decomposition rate of H_2_O_2_. With increased the stabilizer dosage, the polymer chain segments can not stretch freely, and the probability of mutual adsorption and chelation between the curled polymer chains increased, which weakened the adsorption and chelation of the surrounding substances catalyzing the decomposition of H_2_O_2_, and the ineffective decomposition of H_2_O_2_ increased. So the suitable dosages of this stabilizer in the bleaching of linen yarn should be 4 g/L.

#### Effect of stabilizer dosages on whiteness

Different dosages of the stabilizer leaded to different inhibition on the decomposition of H_2_O_2_, which directly affects the effective decomposition or excessive decomposition of H_2_O_2_, and affects the whiteness of the bleached textile materials. Invalid or excessive decomposition of H_2_O_2_ not only affects the whiteness, but also causes fiber demage. The effect of synthesized stabilizer dosage on whiteness was shown in Table 3.

**Table 3:** The effect of synthesized stabilizer dosage on whiteness.

Dosages of the synthesized stabilizer /(g/L)	2	4	6	8
Whiteness/%	64.30 ± 0.15	66.81 ± 0.58	65.50 ± 0.39	61.86 ± 0.51

From the results shown in Table 3, when the bleaching bath contained 4 g/L synthesized OBS, the whiteness of the bleached sample reached the maximum 66.81%. Continued to increase the stabilizer dosage, the whiteness decreased instead. When the stabilizer dosages reached 8 g/L, the whiteness of the bleached sample was down to 61.86%. Because of exceeding 4 g/L of the stalizer dosage, the decomposition rate of H_2_O_2_ increased, resulting in ineffective decomposition, so the whiteness also decreased. Therefore, the most suitable amount of this stabilizer in the bleaching of linen yarn was 4 g/L.

#### Surface examination by scanning electron microscopy (SEM)

**Figure 9:**
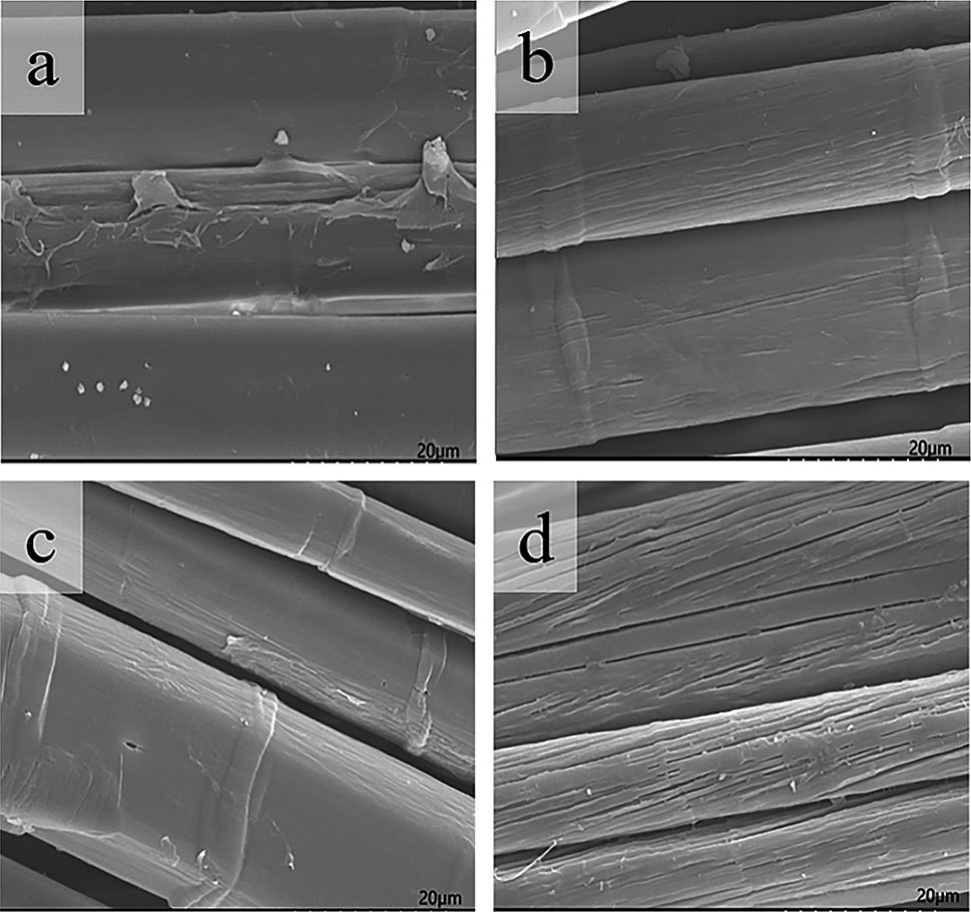
SEM photos of the bleached samples with different dosages of the synthesized OBS. (**a**) 2 g/L OBS; (**b**) 4 g/L OBS; (**c**) 6 g/L OBS; (**d**) 8 g/L OBS.

SEM examination of the linen yarn was made with S-3400 Scanning Electron Microscope by Hitachi Co. (Japan). SEM photos of the bleached samples with different dosages of the synthesized OBS were given in Fig. 9.

It can be seen from Fig. 9, the slight change on the fiber surface was clearly observed. While the stabilizer dosage was 2 g/L (a) , there were granular solids on the fiber surface, which may be the colloidal material existed in the flax fiber itself, or metal ions and impurities chelated by the stabilizer adhesived to the fiber, resulted in poor handle and lower whiteness. While OBS dosage was 4 g/L (b), there were no solid particles on the fiber surface, and the surface was smooth and loose, so it had good handle and higher whiteness, which helped to subsequent dyeing process. While OBS dosage was 6 g/L (c), there was basically no solid substance attached on the fiber surface, and the surface was relatively smooth and loose, with better handle and higher whiteness, but appeared some clear micropores. When OBS dosage was 8 g/L (d), there were less amount of solid particles on the surface of the fiber, and appeared severe cracks on the fiber surface, which maybe inevitably decrease the fiber breaking tenacity.

According to the influences of the above-mentioned dosages of the synthesized OBS on the decomposition rate of H_2_O_2_ and whiteness of the bleached sample, in combination with the analysis of SEM photos, The dosages of the synthesized OBS should be 4 g/L.

## Conclusions

In this study, a non-silicon oxygen bleaching stabilizer, based on acrylic acid and magnesium sulfate, was synthesized for hydrogen peroxide bleaching. The optimal synthesizing process of the stablizer was determined, namely, acrylic acid monomer concentration 25%, initiator potassium persulfate 5% of the mass of acrylic monomer, reacted at 65 °C for 4 h, then added magnesium sulfate (the mass ratio of acrylic acid and magnesium sulfate is 5:1), continued to react for 1 h. The synthesized complex compound of poly(acrylic acid) and magnesium salt had good chelating effect, the chelated iron value reached 239.314 mg/g, and the chelated calcium value reached 145 mg/g, so it can effectively inhibit the rapid decomposition of hydrogen peroxide caused by the presence of metal ions in the bleaching solution. The molecular weight of the polymer was uniformly distributed, ranging from 890 to 1250. The bleaching results indicated that the optimal dosage applied to linen yarn was 4 g/L. the decomposition rate of hydrogen peroxide was no more than 50% in 60 min, the whiteness reached 66.81%. Compared with sodium silicate as the traditional oxygen bleaching stabilizer, the synthesized poly(acrylic acid) magnesium salts complex compound had the similar whiteness performence, and the bleached samples had much better handle and no silicon scale generated, it can effectively solve the silicon scale problem.

This study is expected to provide contribution to the future develop of non-silicon oxygen bleaching stabilizer. For future work, the synthesized poly(acrylic acid) magnesium salts complex compound will be applied for banana and hemp fibers bleaching and the related environmental friendly bleaching processes will be investigated.
